# The equine navicular apparatus as a premier enthesis organ: Functional implications

**DOI:** 10.1111/vsu.13620

**Published:** 2021-03-12

**Authors:** Michelle L. Osborn, Jean Luc Cornille, Uriel Blas‐Machado, Elizabeth W. Uhl

**Affiliations:** ^1^ Department of Comparative Biomedical Sciences School of Veterinary Medicine, Louisiana State University Baton Rouge Louisiana USA; ^2^ Science of Motion Eatonton Georgia USA; ^3^ Department of Pathology College of Veterinary Medicine, University of Georgia Athens Georgia USA

## Abstract

Navicular syndrome has been traditionally characterized by progressive lameness with chronic degeneration of the navicular bone. Advances in imaging techniques have revealed that its associated soft tissue structures are also affected. This distribution of lesions is explained by conceptualizing the equine navicular apparatus as an enthesis organ that facilitates the dissemination of mechanical stress throughout the tissues of the foot. The navicular apparatus has the same structural adaptations to mechanical stress as the human Achilles tendon complex. These adaptations efficiently dissipate mechanical force away from the tendon's bony attachment site, thereby protecting it from failure. The comparison of these two anatomically distinct structural systems demonstrates their similar adaptations to mechanical forces, and illustrates that important functional insights can be gained from studying anatomic convergences and cross‐species comparisons of function. Such a functional conceptualization of the equine navicular apparatus resolves confusion about the diagnosis of navicular syndrome and offers insights for the development of mechanically based therapies. Through comparison with the human Achilles complex, this review (1) re‐conceptualizes the equine navicular apparatus as an enthesis organ in which mechanical forces are distributed throughout the structures of the organ; (2) describes the relationship between failure of the navicular enthesis organ and lesions of navicular syndrome; (3) considers the therapeutic implications of navicular enthesis organ degeneration as a form of chronic osteoarthritis; and based upon these implications (4) proposes a focus on whole body posture/motion for the development of prehabilitative and rehabilitative therapies similar to those that have already proven effective in humans.

## 1 INTRODUCTION

Navicular syndrome is characterized by chronic, progressive lameness associated with caudal heel pain and the degeneration of the navicular bone and its surrounding structures. Over time, the specific name for this syndrome has changed from navicular disease to caudal heel pain, to palmar foot pain, to podotrochlosis/podotrochlitis, as characterization of the condition has developed through advances in imaging and the assessment of lesions in both the bone and soft tissues. Additionally, “navicular disease” has become a loaded term associated with a poor prognosis.^1^ “Podotrochlosis” represents the current understanding that the damage characterizing navicular syndrome affects the entire podotrochlear apparatus. However, we will continue to use “navicular syndrome” and “navicular apparatus,” as these terms are the most familiar to horse owners, trainers, and veterinarians.

Navicular syndrome most often affects Quarter horses, Thoroughbreds, and European Warmbloods, and is usually diagnosed in geldings and stallions between 4 and 15 years in age, during various stages of training and work.^2^, ^3^, ^4^ Because it affects different breeds and horses with varying foot conformations, navicular syndrome is thought to have an important biomechanical component and to be related to the way in which a horse moves or is being worked.^2^, ^3^, ^4^ Clinically navicular syndrome is characterized by forelimb lameness that is usually bilateral in presence but asymmetrical in severity. Diagnostic procedures focus on isolating the navicular region as the source of the pain while also ruling out other potential issues.^4^ Clinical examinations consist of identifying the presence of heel pain with the use of hoof testers, especially in the middle third of the frog, or with the use of extension, flexion, and/or wedge tests.^4^ Further tests that confirm the diagnosis may include injection of anesthetics to block the palmar digital nerves, the distal interphalangeal joint, or the navicular bursa along with diagnostic imaging such as radiography, X‐ray computed tomography (CT), magnetic resonance imaging (MRI), and potentially bursoscopic examination.^2^

### 1.1 The role of soft tissue imaging in the diagnosis of navicular syndrome

In general, our understanding of navicular syndrome is limited not only by how we are able to study it but also by how we understand the relevant anatomical structures. Recent advances in technology such as bursography, ultrasonography, and MRI have enabled the visualization of soft tissues. In addition, advances in documenting the cellular and physiological changes occurring within this area have been enabled by nuclear scintigraphy, thermography, and the study of synovial fluid biomarkers. Thus, our understanding of navicular syndrome has changed from a belief that the bone was the central affected structure, to a recognition that multiple structures within the area are also affected.^2^, ^5^ It should be noted, however, that the soft tissue structures damaged in navicular syndrome are not always examined at necropsy as the diagnostic emphasis in pathology has generally remained on the bone lesions. This persistent focus on bony structures may originate with a traditional view of anatomy that is underpinned by comparisons with architecture and engineering.

Advances in imaging technology are helping us to replace this faulty image and see the way that soft tissue structures maintain posture and enable movement in living animals. In navicular syndrome, improvements in imaging have allowed for earlier detection of tissue damage and led to better characterization of individual lesions.^5^, ^6^, ^7^, ^8^ However, more detailed imaging has also complicated the diagnosis by identifying the presence of lesions in multiple tissues associated with the navicular bone.^5^, ^6^, ^7^, ^8^ The question is whether these lesions represent different disease processes or result from the failure of a functional complex: in this case, the navicular enthesis organ. Considering the structures in the foot to be an enthesis organ^9^ is clinically useful, as it explains why the lesions of navicular syndrome affect multiple structures and vary between cases.

### 1.2 Historical versus current perspectives and treatments

Current nonsurgical treatments for navicular syndrome include rest, controlled exercise under saddle, hoof trimming designed to level or balance the foot, especially by providing heel support, and therapeutic shoeing specific to the individual animal (although opinions of the type of shoeing that should be used have varied).^10^ These interventions have generally been unsuccessful over the long term and it has become necessary to investigate other therapies.^5^, ^11^

Pharmacologic interventions geared toward reducing pain and inflammation include injecting corticosteroid or glycosaminoglycan into the joint, bursa, or tendon sheath of the deep digital flexor muscle, or administering non‐steroidal anti‐inflammatory drugs (NSAIDS), vasodilators, and nutraceuticals geared toward overall joint health. Because resorption of the navicular bone is often found in horses with navicular syndrome, various bisphosphonates (e.g., tiludronate) are used to normalize the bone‐remodeling unit by inhibiting bone resorption by osteoclasts.^12^, ^13^, ^14^ Some of the commonly used pharmacologic interventions, namely anti‐inflammatory drugs, have been criticized for masking the pain associated with athletic injury, leading to overexertion, more tissue damage, and catastrophic breakdown, especially in racehorses.^15^

Surgical treatment is generally reserved for the most severe cases after other treatments have failed; however, postoperative issues are relatively common. Desmotomies are used to try and re‐position the navicular bone itself, but instability of the distal interphalangeal joint is of concern. Neurectomies designed to desensitize the area may lead to neuromas and rupture of the tendon of the deep digital flexor muscle.^5^ In some clinics, both of these surgical treatments are being replaced by bursoscopically facilitated treatments including bursal lavage and debridement of damaged soft tissue (e.g., tendon of the deep digital flexor muscle) and/or hard tissues (e.g., navicular bone).^6^, ^16^, ^17^

Over time, horses often become unresponsive to all of these treatments, which are palliative rather than curative, and affected animals are thus considered to have an incurable, progressive disease.^5^, ^18^ Cutting edge regenerative therapies using, for example, stem cells, bone marrow, or growth factor^19^, ^20^, ^21^, ^22^, ^23^, ^24^, ^25^, ^26^, ^27^ are being used to manage the disease by trying to establish an equilibrium between the damage or loss of tissues and their regeneration, aiming to limit the progression of tissue degeneration. Successes in early stem cell research into therapies in veterinary medicine (notably in the horse and dog) have provided the translational basis into human regenerative medicine.^28^ While these therapies aim to modify the disease process^29^ and short‐term outcomes appear favorable,^30^ the long‐term outcomes, especially in the face of ongoing mechanical overloading, are largely unknown.^31^ Still, the promise of favorable outcomes^32^ has led to the implementation of this therapy.

Despite numerous excellent studies of the lesions, their pathogenesis has been, as James Rooney put it: “long, assiduously, and fruitlessly debated.”^33^ Part of the problem is the lack of direct correlation between the manifestation of lameness and the structural lesions; horses can be lame with no lesions, or sound with lesions. The general consensus is that, while the pathogenesis of navicular syndrome is likely to be multifactorial, it is mechanically related,^3^, ^5^ and the degenerative processes involved are similar to those of osteoarthritis.^2^ Although the importance of considering the structures associated with the navicular bone as an inter‐dependent functional complex has been recognized,^2^, ^3^, ^5^, ^8^, ^34^, ^35^, ^36^, ^37^ many therapies still focus on treating specific lesions rather than restoring function to the whole complex.

## 2 ENTHESIS ORGANS

The tiny foot of the horse supports a relatively large body. Failure of such a setup is possible and perhaps even probable, especially when it is subjected to excessive mechanical forces in domestic environments. A main function of the navicular enthesis organ is to enable the internal soft and hard tissues of the foot to withstand impacts of body weight while also permitting various movements. While the interaction of muscle with bone has long been investigated,^38^, ^39^ with studies focusing on development, biomechanical function, and failure,^40^ this substantial field of literature is outside the purview of this review. But to understand the importance of conceptualizing the navicular apparatus as an enthesis organ, some definitions are useful.

### 2.1 Enthesis and functional enthesis

An *enthesis* is the site of attachment of tendon to bone, through which muscular forces are transmitted along tissues with distinct physical properties (i.e., soft, flexible tendons, firmer ligaments, and joint capsules and hard bone). The presence of fibrocartilaginous tissues within this transition from tendon to bone is evidence that there are large forces acting on an enthesis, and any enthesis that contains fibrocartilage is particularly vulnerable to damage.^9^, ^41^ The term *functional enthesis* refers specifically to situations in which tendons or ligaments wrap around bone proximal to their actual attachment sites, creating a pulley system that disperses forces before they reach the junction of soft tissue to bone.^42^, ^43^

### 2.2 Enthesis organ and synovio‐entheseal complex

**FIGURE 1:**
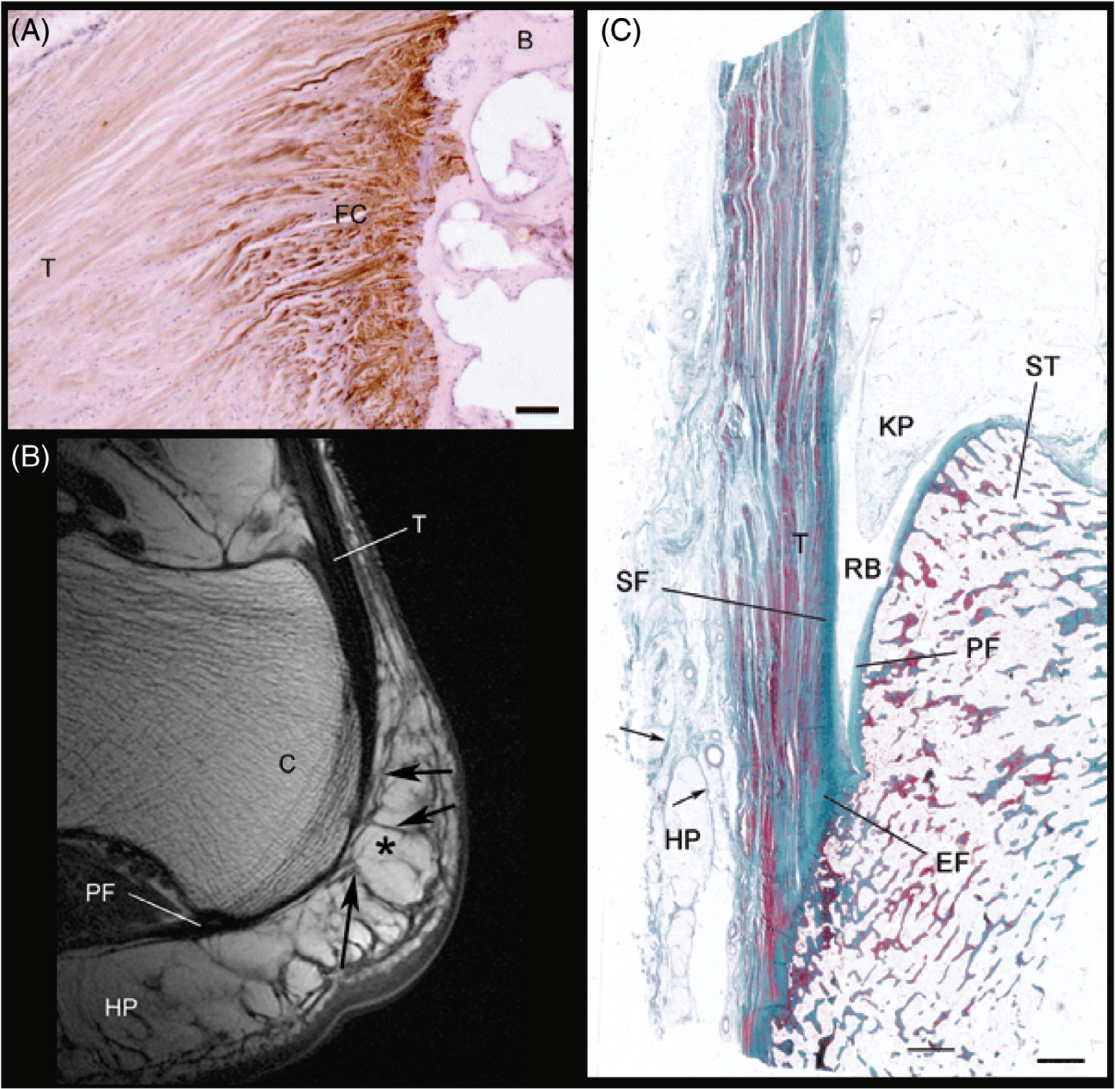
Human enthesis organ structures (reproduced with permission from John Wiley and Sons). (A) From Benjamin et al. 2006, figure 3^9^: Fibrocartilaginous enthesis of the extensor tendon on the middle phalanx of the second toe. B: bone, FC: fibrocartilage (immunohistochemically labeled), T: extensor tendon. Scale bar: 100 um. (B) From Shaw et al. 2008, figure 2^44^: Adult female human Achilles tendon enthesis organ, sagittal MR image: T: Achilles tendon, PF: plantar fascia, C: calcaneus, HP: heel fat pad. Asterisk: fat, arrows: fibrous septa. (C) From Shaw et al. 2008, figure 1^44^: The human adult Achilles tendon enthesis organ. EF: enthesis fibrocartilage, SF: sesamoid fibrocartilage, PF: periosteal fibrocartilage, ST: superior tuberosity of the calcaneus, T: Achilles tendon, RB: retrocalcaneal bursa, KP: Kager's fat pad, HP: Heel fat pad. Arrows: fibrous septa within the heel fat pad. Masson's trichrome. Scale bar: 2 mm

An *enthesis organ* comprises the actual enthesis, joint capsules, ligaments, and bones, along with the adjacent fibrous and adipose tissues (Figure 1),^9^, ^44^, ^45^ including the as yet un‐named fibrous connections between these various structures.^45^ Thus, mechanical stress transmitted by tendons not only affects the enthesis but also the surrounding tissues, as it is dissipated away from the enthesis itself. This concept of an enthesis organ provides the foundation for understanding the varied location of the mechanically induced lesions characteristic of navicular syndrome. These result not just as forces pass from tendon to bone but also as they pass throughout fascial connections to the various structures involved.^9^, ^41^, ^45^, ^46^ A causal relationship between microdamage of the enthesis and the induction of inflammation in the synovial membrane is expressed by the term *synovio‐entheseal complex* (SEC).^45^, ^47^

**FIGURE 2:**
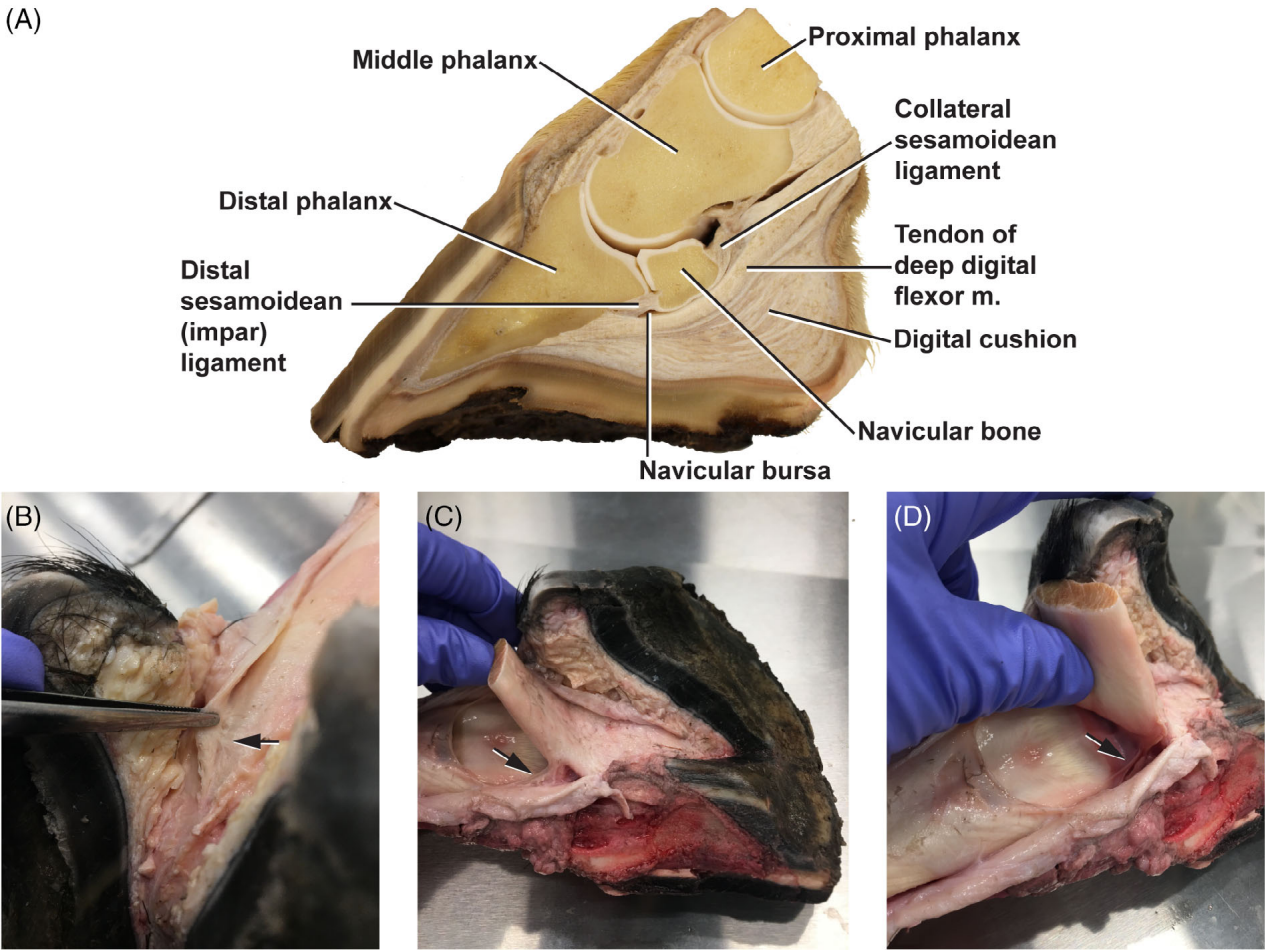
The equine navicular enthesis organ. (A) Structures of the navicular apparatus. Fascial connections (arrows) between: (B) Distal digital annular ligament and DDFT.^46^ (C) Superficial and deep digital flexor tendons. (D) DDFT and navicular bone^45^

**FIGURE 3:**
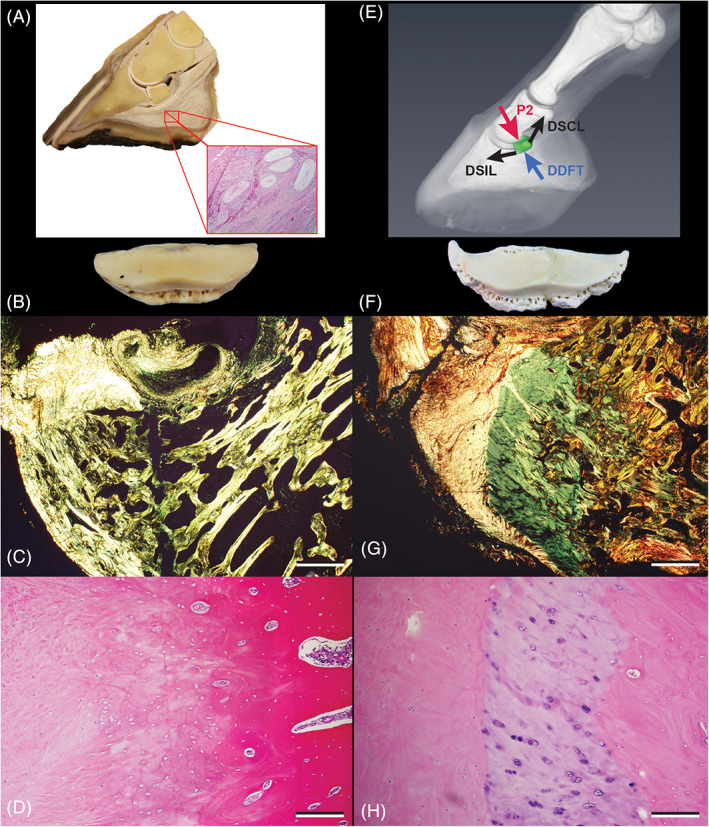
The equine navicular enthesis organ and common lesions. (A) The equine navicular enthesis organ. Pacinian corpuscles (inset) near the navicular bursa. (B) Navicular bone and (C) and (D) DSIL enthesis from an adult Quarter horse gelding with no clinical or pathological evidence of navicular syndrome. Polarized picrosirus red (PR) (C) and hematoxylin and eosin (H&E) (D) stained histological sections. The unaffected enthesis has mostly type 1 collagen fibers (yellow in C) that blend smoothly with those of the bone (D). (E) Image of reconstructed 3D data of an equine distal limb with force vectors added. DSIL: Distal sesamoidean impar ligament; DSCL: distal sesamoidean collateral ligament. (F) Navicular bone and (G) and (H) DSIL enthesis from an adult Paint horse euthanized for navicular syndrome. Polarized PR (G) and H&E (H) stained histological sections. Bony enthesophytes are at the attachment sites of the DISL and collateral ligaments (F). Cartilage present at the DISL enthesis (green in G) and disrupts the smooth transition between ligament and bone (light blue in H). (C) and (G) = 20X, scale bar: 500 μm; (D) and (H) = 100X, scale bar:100 μm

**FIGURE 4:**
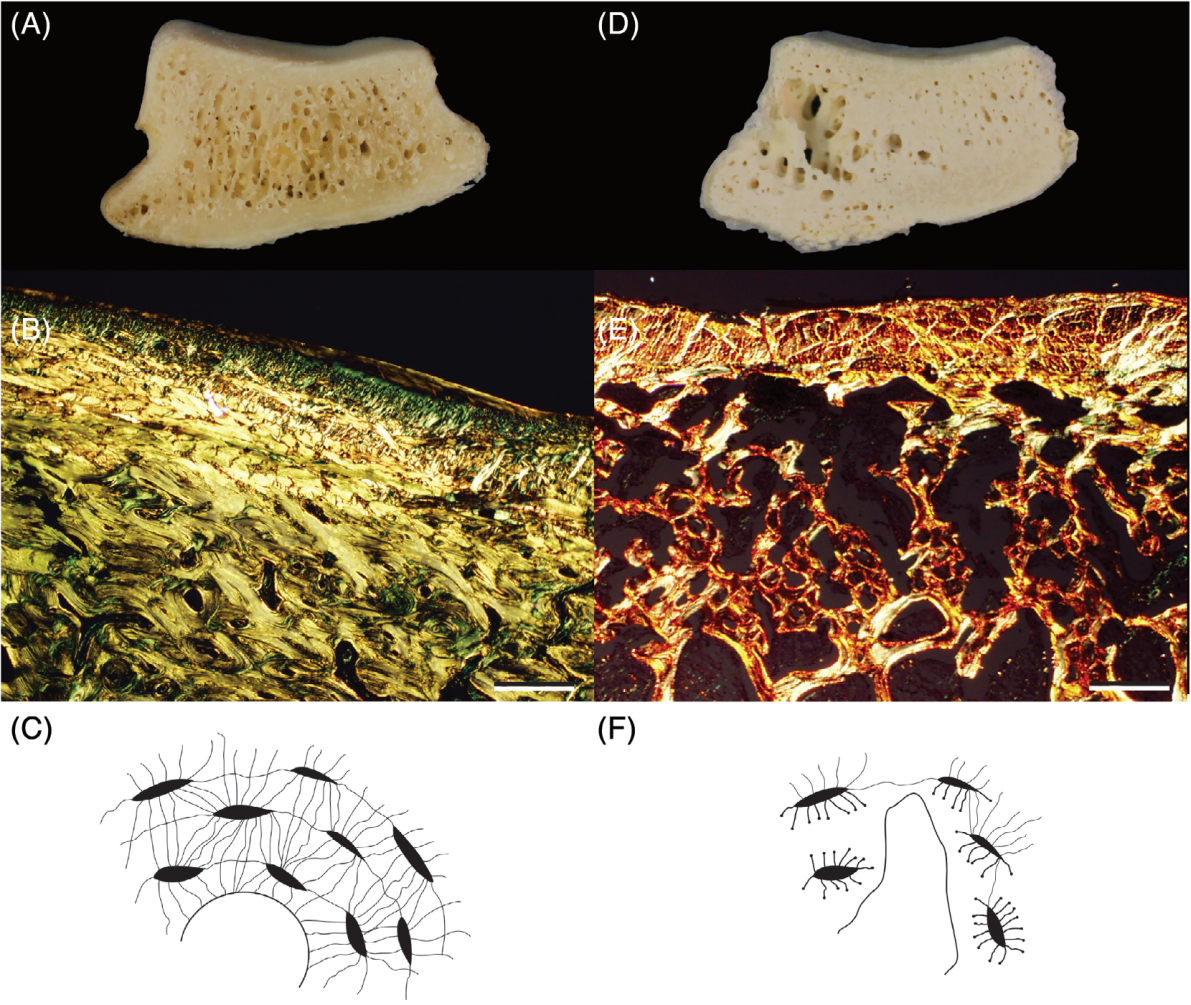
Common bone lesions in navicular syndrome. Navicular bone from an adult Quarter horse gelding with no clinical or pathological evidence of navicular syndrome (A) Cross‐section. (B) Orderly collagen fibers within the fibrocartilage and subchondral bone: palmar surface. PR. (C) Diagram of normal osteocyte network. Navicular bone from an adult Paint horse euthanized for navicular syndrome (D) Cross section showing bone loss and irregular palmar surface. (E) Disrupted collagen fibers, loss of fibrocartilage and subchondral bone: palmar surface. PR. (F) Diagram of osteocyte loss and network disruption.^50^ (B) and (E) = 40X; scale bar: 200 μm

**FIGURE 5:**
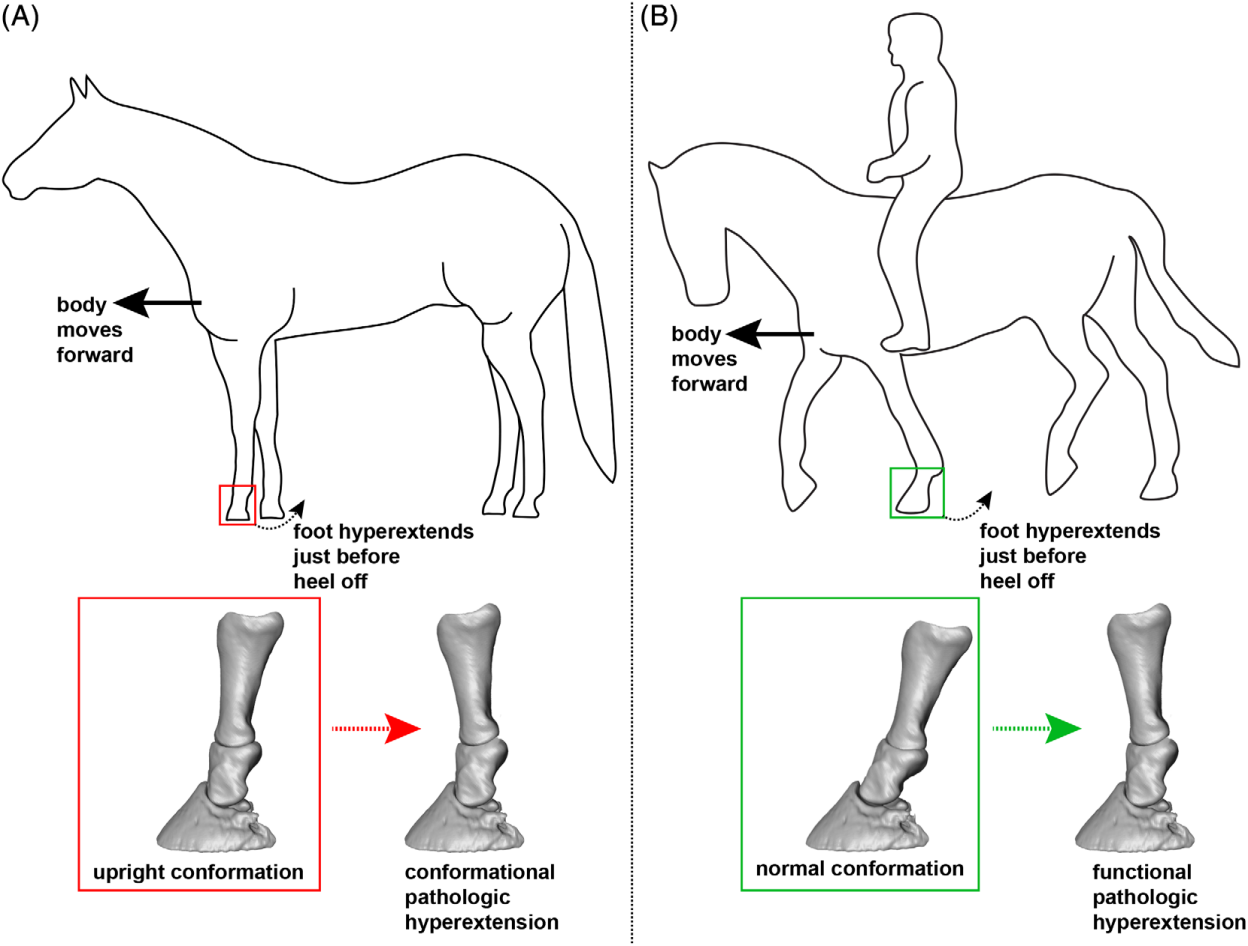
Conformation and posture/movement in navicular syndrome. (A) Tracing of a Quarter horse with a large body, small foot, and upright distal limb conformation that can predispose to navicular syndrome; this tracing, which enables form to be observed while also removing identifying features, was done on an image from the internet. As this horse moves forward, the foot becomes hyperextended just before heel‐off, creating a pathological position of the foot that compresses the structures of the navicular enthesis organ. (B) Tracing of an 11‐year‐old Warmblood with navicular disease and a normal foot conformation at the trot; this tracing of the common working posture was done on a representative still image from videos of the horse. This horse is poorly balanced, as its body is tipped onto its forehand. As it moves forward, the propulsive phase in which the foot lifts off the ground is delayed, occurring behind rather than in front of the rider's leg. This causes the leg to pass the vertical and become hyperextended just before heel off, creating a pathological position of the foot that has been shown to compress the structures of the navicular enthesis organ^51^

The conceptualization of the navicular apparatus as an enthesis organ links the structure and function of its constituent elements and provides a context of their failure. Within the horse foot, the navicular enthesis organ is made up of a large number of structures including ligaments (collateral sesamoidean ligaments, distal sesamoidean ligament, distal digital annular ligament, and chondrosesamoidean ligaments), tendon of the deep digital flexor muscle (DDFT), digital cushion, joint capsule, navicular bursa, bones: distal phalanx (P3), navicular (distal sesamoidean bone), and distal ½ of the middle phalanx (P2), the medial and lateral ungual cartilages of the distal phalanx, the fibrous enthesis itself, and fascial connections between the various structures (Figure 2).^37^, ^48^, ^49^ All of these can be damaged by the mechanical forces impacting the DDFT, the intensity, and direction of which are determined by the positioning of the body and the leg. In particular, hyperextension of the distal limb produces compression of the bone by the DDFT (Figures 2, 3, 4, 5). This also occurs in dorsiflexion of the human ankle, which is equivalent to hyperextension of the distal limb in quadrupeds. Consideration of the forces acting on these structures and how they are adapted to function as an enthesis organ provides a better understanding of the pathogenesis of navicular syndrome as well as an explanation for the variation in its clinical presentation.

Even though they are not direct evolutionary homologies, the human Achilles tendon apparatus, often cited as the “premier enthesis organ,”^52^, ^53^, ^54^ and the equine navicular apparatus have similarities in both structure and function. Importantly, the same type of movement (dorsiflexion/hyperextension) causes similar tissue degeneration within both enthesis organs.

It has been established that in the Achilles enthesis organ, tensile mechanical stress, especially that created by dorsiflexion of the foot, is transmitted away from the actual attachment site of the Achilles tendon by a series of related structural adaptations (Figure 1)^41^, ^44^, ^45^, ^52^, ^53^, ^54^:

1. The Achilles tendon bends around the superior tuberosity of the calcaneus proximal to the enthesis.
2. Fibrocartilage reinforces the tendon at this location.
3. A bursa is positioned between the bone and the tendon.
4. The tendon transitions from dense connective tissue to fibrocartilage to bone.
5. Fat pads are present between the tendon and bone and at the heel of the foot.

The equine navicular apparatus has the same structural adaptations to mechanical stress (Figure 2):

1. *Contact of the respective tendon with bone proximal to the actual enthesis*. The DDFT contacts the navicular bone proximal to its insertion on P3. This creates a functional enthesis in which the DDFT bends around the navicular bone like a pulley, thus maintaining the angle of attachment throughout various movements of the foot (Figures 2 and 3). The navicular bone acts as a fulcrum, providing a long lever arm and thus mechanical advantage, for the action of the DDFT on P3 (interestingly, the navicular bone is fused to P3 in the more massive rhinoceros, producing a more stable fulcrum for the DDFT; personal observation). This bony contact protects the actual enthesis when mechanical forces are transferred from the tendon to the navicular bone before the actual insertion site.^42^, ^43^ However, excessive forces will increase pressure on the bone and cause damage, usually manifesting initially as edema. Bone edema is also observed in the superior tuberosity of the calcaneus in humans, which has a similar function as a fulcrum, with increased stress on their Achilles tendon.^55^
2. *The presence of fibrocartilage at the site where the tendon bends around the bone*. Fibrocartilage, both within the DDFT and on the palmar surface of the navicular bone where it contacts the tendon,^56^ is an adaptation to withstand compression and shear, as the extracellular matrix of the fibrocartilage allows for a high water content, which resists compression of the tendon as it passes over the bone.^41^, ^45^
3. *The presence of a bursa between the bone and the tendon*. The navicular bursa has a connective tissue capsule and no communication with the distal interphalangeal joint.^56^ It minimizes frictional forces on the fibrocartilage as the tendon passes over the bone during movements of the foot.
4. *A transition from the dense connective tissue of the tendon to fibrocartilage to bone*. The DDFT has a fibrocartilaginous enthesis in which the tendon transitions from dense connective tissue first to uncalcified, then calcified fibrocartilage as it flares to attach to P3.^57^
5. *The presence of a fat pad*. The digital cushion is much more than a simple fat pad, since its predominant constituent elements are elastic fibers, connective tissue or fibrocartilage, and a gelatinous myxoid matrix, functions as a hydrostatic cushion to minimize pressure changes in the navicular apparatus during movements of the foot;^50^, ^58^, ^59^ it also contains pain and pressure receptors^50^, ^56^, ^60^, ^61^ that can monitor loading of the enthesis (Figure 3).
6. *The attachment of a tendon into a depression*. The navicular enthesis organ has an additional feature that, while present in other enthesis organs, is not prominent in the Achilles: the DDFT attaches into a depression within P3. This facilitates the dissemination of mechanical stress away from the enthesis by providing an additional ridge of bone, a second fulcrum, just before the insertion site. The presence of two specialized structures for the dissemination of mechanical stress within this enthesis organ indicates that it is under an extremely high level of mechanical stress.

Additional adaptations in the horse include the ligamentous structures where cartilage and bone can form in response to mechanical stress, and the fascial connections through which forces are dispersed between structures; for example, between the superficial and deep digital flexor tendons (SDFT & DDFT) or between the DDFT and navicular bone (Figure 2).^48^, ^49^, ^58^ The equine navicular apparatus thus includes all the components of a “premier enthesis organ.” Given that avulsions at the actual enthesis are uncommon in horses,^37^ one could argue that it is even more highly adapted and effective than the human Achilles enthesis organ.

## 3 EXPLAINING THE LESIONS: FAILURE OF THE NAVICULAR ENTHESIS ORGAN

Although their pathogenesis has been debated, a consistent set of lesions is reported in horses with navicular syndrome (Figures 3 and 4).^2^, ^57^, ^62^, ^63^, ^64^, ^65^ The disease has classically been associated with degeneration, sclerosis, and bone loss in the navicular bone, all of which are compatible with a failure to adapt to the mechanical forces, specifically compression, impacting the bone.^62^, ^63^, ^64^, ^65^ A motion analysis study found that horses with navicular syndrome had almost twice the force and stress levels on the navicular bone as sound horses; this increased compression of the navicular bone was at levels known to cause cartilage damage.^66^ Another study found that lesions occurred in areas on the palmar surface of the navicular bone that were exposed to the maximum pressure of the tensed DDFT, ^67^ confirming the association of compression with specific sites of tissue damage. Although the severity of the lesions varies, they are typically present throughout the bone and consist of chronic degeneration and extensive but ineffective bone remodeling with focal osteolysis in the palmar cortex, punctate erosions in the medullary trabeculae, generalized loss of bone mass, microfractures, and fibrosis (Figure 4).^62^, ^63^, ^65^, ^68^ These changes are caused by levels of mechanical stress that are beyond the tissue's ability to adapt to it.

Bone remodeling is critical to repair mechanically induced damage and there is a question of why it is not effective in cases of excessive compression. The adaptation of the bone to increased loading is dependent on the presence of normal osteocytes. However, navicular bones from diseased horses had reduced osteocyte densities and extensive loss of osteocyte connectivity, especially in areas having the greatest cyclic loads, such as the distal border, mid‐sagittal section, and flexor surface.^65^ Osteocytes sense mechanical stress by compression of their processes and are responsible for inducing and directing the repair response.^65^ Therefore, the combination of decreased numbers and disruption of the osteocyte network by continued compression would explain the ineffective remodeling observed in navicular syndrome (Figures 3 and 4).^65^

Histopathological studies and recent advances in imaging have demonstrated that many of the soft tissue structures surrounding the navicular bone, including the navicular bursa, deep digital flexor tendon, and the distal sesamoidean impar ligament (impar; DSIL) and collateral sesamoidean (collateral) ligaments also have degenerative lesions in horses with navicular syndrome.^8^, ^35^, ^36^, ^37^, ^69^, ^70^ In particular, increased tension on the DDFT results in compression of both the navicular bone and the DDFT itself but is also transmitted through fascial connections between various structures including the impar and collateral ligaments, leading to their degeneration and the development of enthesophytes (osteophyte‐like bony projections formed at an enthesis in response to stress) (Figure 3).^37^, ^48^, ^49^, ^71^, ^72^

The observations that all of the structures damaged in horses with navicular syndrome are components of the navicular enthesis organ, and the type of damage they display is compatible with mechanical compression, indicate the lesions are induced by the failure of this organ to effectively disperse the mechanical forces impacting the foot. In addition, understanding the synovio‐entheseal complex (SEC) and the “functional enthesis” concepts helps in understanding the relationship between mechanically induced degeneration and inflammation in the navicular enthesis organ, particularly in the bursa. The SEC concept explains the interplay between the synovial membrane and the enthesis. Based on their cellular components, the enthesis appears to be anti‐inflammatory and the synovium pro‐inflammatory. Thus, the microdamage to which the enthesis is especially susceptible stimulates an inflammatory reaction in the synovium.^47^ The ability of mechanical forces to directly stimulate gene expression of inflammatory mediators through mechanotransduction^73^, ^74^, ^75^, ^76^ is an area ripe for further study in navicular syndrome. The implication of the direct up‐regulation of inflammatory mediators by mechanical forces is that a vicious cycle can be set up in which treatment with anti‐inflammatories allows a horse to continue to move in a way that induces pathological mechanical stress and more inflammation; thus, ever higher doses of anti‐inflammatory drugs are needed until eventually the lameness is unresponsive. The role of mechanics and microdamage in the upregulation of angiogenesis is another area worth investigating, as a characteristic feature of navicular syndrome is vessel ingrowth into the normally avascular fibrocartilage.^65^

In summary, the lesions that characterize navicular syndrome are compatible with severe chronic mechanical compression that damages ligament attachment sites as well as the bone and inhibits repair responses. The disease often has a poor prognosis for returning to soundness^1^ and will continue to progress unless the source of the mechanical compression is identified and relieved. As would be expected, if the source of the compression is extrinsic (i.e., overloading of the whole enthesis organ), rather than intrinsic to the bone, the surrounding soft tissue is also extensively damaged (Figures 2 and 3). While advances in imaging have led to earlier detection of the soft tissue damage characteristic of navicular syndrome, they have also led to more confusion about exactly how to define the disease. Most of this confusion would be resolved, as it has been for other diseases, if the definition of navicular syndrome was based on its primary cause: mechanical overloading of the foot, rather than on the fine details of the resulting tissue lesions.

## 4 MECHANICAL STRESS, PAIN, AND TISSUE INJURY

The location of pressure and pain receptors provides additional evidence that the navicular apparatus functions as an enthesis organ. The ability to sense insults that cause tissue damage before the damage occurs has evolutionary advantages and is characteristic of certain types of pain sensors (i.e., those for heat, cold). The well‐developed ability to sense mechanical stress before it causes significant tissue damage should not be surprising, given the critical importance of locomotion to an animal's survival. Pain must therefore be investigated as an important indicator of pending, as well as present, tissue damage.^77^ In horses, Pacinian corpuscles, which are mechanoreceptors adapted to sense pressure, are most common in the caudal aspects of the heel, in close proximity to the navicular bursa, and in the suspensory ligaments of the navicular bone, all of which as parts of the navicular enthesis organ are subjected to high mechanical forces (Figure 3).^56^, ^60^ Although Pacinian corpuscles are not traditionally considered to be pain receptors, in the human hand hypertrophy and hyperplasia of these corpuscles has been associated with pain as well as a history of trauma.^78^ In addition, along with nociceptors, Pacinian corpuscles have been identified in the adipose tissue of other entheses and are present in increased numbers within the fibrocartilage masses at sites of spondylosis.^44^, ^79^ These observations suggest that mechanoreceptors sense instability at sites under mechanical stress, and that nociceptors then convey this as pain in order to elicit a protective response.^79^ Whether Pacinian corpuscles may play a similar role in navicular syndrome still needs to be investigated. In the meantime, *pain as an indicator of pending mechanical tissue damage* helps explain another enigma of navicular syndrome: why horses that have only mild or even undetectable lesions display the lameness characteristic of navicular disease.

For these cases, veterinary practitioners might take advantage of an approach used in human medicine. In dancers for example, pain not yet associated with detectible tissue lesions is treated as a pending injury and necessitates a mechanical assessment of their whole body function.^77^ Significantly, this often reveals that the primary biomechanical dysfunction is distant from the site of pain.^77^ For example, insufficient external rotation of the hip is often an important cause of knee and ankle pain and injuries, even though pain and injury to the hip itself are relatively rare.^77^ Doctors treating dancers have thus learned to assess critical components of their technique, such as the “turn‐out” of the hip, especially in cases where pain is present but lesions have not yet developed.^77^

## 5 NAVICULAR ENTHESIS ORGAN DEGENERATION: INSIGHTS AND THERAPEUTIC IMPLICATIONS FROM CHRONIC OSTEOARTHRITIS

Both navicular syndrome and chronic osteoarthritis or degenerative joint disease have a mechanical pathogenesis and, at the tissue level, the lesions are virtually identical. For example, the lesions that commonly occur in the bone, at the interface between the cartilage and subchondral bone and at the impar and collateral ligament attachment sites, in navicular syndrome are the same as those described in chronic osteoarthritis (Figures 3 and 4).^51^, ^64^, ^65^, ^67^, ^80^, ^81^, ^82^ Because of these similarities, consideration of navicular syndrome as a form of chronic osteoarthritis can provide useful insights about both the features of the disease and effective therapies for treating it. For example, one of the confusing aspects of navicular syndrome, also found in other forms of osteoarthritis, is that the lesions do not always correlate with the degree of lameness; a phenomenon which is also observed in cases of human osteoarthritis.^83^ In both diseases the lack of pain, even when lesions are present, can potentially be explained by whether the mechanical stress is actively occurring at the time the patient presents. Another factor is whether the lesions have provided effective compensation for increased mechanical stress. For example, the formation of cartilage and bone (enthesophytes) in the attachments of tendons, joint capsules, and ligaments (i.e., the DSIL as shown in Figure 3), although considered pathologic is also a compensation that reinforces and stabilizes these attachment sites against the mechanical forces impacting them.^84^

In addition to the similarities in clinical presentation and pathology, the traditional treatments for chronic osteoarthritis in humans are basically the same as those used for equine navicular disease: rest, pharmacotherapy, localized external support, restriction/redirection of movement of the affected area (i.e., through footwear and braces vs. hoof trimming and corrective shoeing) and various surgical interventions. In human medicine, frustration with the ineffectiveness of these treatments, along with major advances in sport/occupational medicine, has led to an increased focus on the role of whole‐body mechanics. As a result, more effective physical therapies for osteoarthritis have been developed based on a comprehensive as opposed to localized understanding of the mechanics of movement. Prehabilitative and rehabilitative therapies that begin with examining an individual's posture and movement are now widely accepted as effective in the prevention and treatment of human athletic and work‐related injuries.^83^, ^85^, ^86^ Although therapies of this sort have been considered for horses,^8^ they have not been systematically adapted for the prevention and treatment of equine lameness. For the most part, veterinary practitioners have continued to focus on tissue lesions rather than on the aberrant whole‐body mechanics that created them.

Importantly, biomechanical analyses of human athletic injury and chronic work‐related cases of osteoarthritis have found that the primary site of mechanical dysfunction is often not at the site of lesion development.^77^ Several studies in horses have concluded that compression initiates and promotes degenerative changes in navicular syndrome. In particular, they found excessive forces impacting the distal half of the flexor surface of the navicular bone are often sustained, resulting in increased pressures at levels known to cause pain in humans.^62^, ^63^, ^64^, ^65^, ^66^, ^67^ However, these studies did not go on to specifically identify the source of the pathological forces.^62^, ^63^, ^64^, ^65^, ^66^, ^67^ While it was proposed as early as 1982 that the disease should be reversible if the excess load on the navicular bone was relieved,^62^ the focus has remained on detailing the lesions and the biomechanics of the distal limb, rather than tracing the mechanical forces causing the overloading back to their origin. Except for poor conformation of the forelimbs, a risk factor present in horses that do and do not develop navicular syndrome,^63^, ^66^ the primary mechanical causes of navicular syndrome have not been systematically investigated.

The fundamental importance of whole‐body biomechanics is that it explains both the location and character of the lesions and why, in navicular syndrome as in chronic osteoarthritis, therapeutic approaches based only on pharmacotherapy and/or surgical intervention generally fail to prevent the recurrence and progression of disease.^87^ In contrast, for human osteoarthritis patients, the use of mechanical therapies based on the analysis of an individual's whole‐body posture and movement is effective in both treating the pain and preventing the disease from progressing no matter how extensive the lesions.^87^, ^88^, ^89^ In an exploratory study using human individuals with osteoarthritis lesions in the knee with a range of severity (i.e., Kellgren/Lawrence grades 0–4), significant improvements in movement, function, pain, and muscle strength were noted in all groups after 12 weeks of exercise therapy. Even the most extreme cases improved to some degree, although they did not show as large a response for certain parameters.^90^ Individualized physical rehabilitative therapy has also improved surgical outcomes and has often made drug treatments and further surgical interventions unnecessary.^87^

## 6 TOWARD THE DEVELOPMENT OF INDIVIDUALIZED MECHANICS‐BASED THERAPY FOR HORSES: PRELIMINARY OBSERVATIONS ON THE EFFECTS OF CONFORMATION AND POSTURE/MOVEMENT ON MECHANICAL LOADING OF THE NAVICULAR ENTHESIS ORGAN

The success of mechanically based therapies in treating human musculoskeletal injuries provides a precedent for extending this approach to the treatment of horses. As previously mentioned, physicians treating ballet dancers routinely assess their dance technique in order to determine whether the injury in question was the result of poor posture in movement, as is commonly the case. In treating dancers, these physicians must have a working knowledge of dance movements and an awareness of common postural problems and their consequences—such as that the inability to maintain full external rotation of the hips increases stress on the knees and ankles.^77^ A similar whole body approach has led to effective biomechanical treatments for damage to the Achilles enthesis organ.^91^, ^92^ These include: strengthening of specific muscles, varying exercise between speed and level of impact, carefully selected exercise surfaces, incremental increases in training, and weight loss.^91^, ^92^, ^93^

As with humans, the starting point for designing effective biomechanical therapies for horses with navicular syndrome is an ability to recognize abnormalities not just in conformation, but in habitual body posture. Unlike conformation, working body posture is often not considered clinically, but has a major impact. It can even explain why some horses with at‐risk conformation never develop navicular disease, while many with good conformation have progressive disease. While conformation can predispose to the overloading of the navicular enthesis, especially in horses with a large body, an upright leg conformation, and very small feet (Figure 5(A)), the defects, unless extreme, can potentially be compensated for by good posture, especially in movement.^94^, ^95^, ^96^, ^97^ In contrast, as illustrated by the case shown in Figure 5(B), poor posture in movement can over‐ride the protective effects of good body and limb conformation. In these cases, it is critical to understand that postural overloading can be caused by a primary dysfunction at a site distant from the navicular enthesis.

The case example in Figure 5(B) illustrates how poor whole‐body posture/movement can lead to the pathological hyperextension of the foot, a position that compresses the navicular enthesis organ^51^ even in a horse with good limb conformation. The outline shows the common working posture of an 11‐year‐old Warmblood gelding at the trot. He was diagnosed with navicular syndrome based upon clinical exam, radiographic findings, and the results of nerve blocks. When compared to the horse in 5A, it is apparent that the trunk of the poorly balanced horse in 5B, rather than being level, is tipped forward, thus overloading the forelimbs. This body posture has resulted in a forelimb kinematic characterized by rotation of the cannon bone (metacarpal III) ahead of the vertical before the foot leaves the ground. This is apparent in the outline by the foot being on the ground behind rather than in front of the rider's leg, which is where it would be if the horse were moving with a balanced body posture (Figure 5(B)). Manipulation of dissected skeletal specimens and models revealed that this kinematic includes hyperextension of the distal interphalangeal joint, which has been experimentally confirmed to maximally compress the navicular bone and associated structures (Figure 5).^51^

In this case, correction of the postural imbalance resolved the horse's chronic lameness and he remained sound even when returned to full work (no therapies other than balanced shoeing were used). This correction was primarily achieved through slowing down the movement until there was a slight pause between the placements of each foot at the walk and improved synchronization of the trot. Such slow movement causes the contractions of the spinal and the extrinsic muscles that attach the thoracic limb to the thorax to become isometric, thereby building up strength with little risk of damage to joints and soft tissues.^98^ Building muscles in this way compensated for loading forces, which led to elevation of the thorax, improved body balance, and thus correction of the forelimb kinematic abnormality that was compressing the navicular enthesis.

Although more research needs to be done, this example demonstrates how a whole‐body approach to the assessment of a mechanically based degenerative disease like navicular syndrome can be practically applied to horses. It also indicates the potential of this approach for the development of individualized motion‐based therapies. Ideally as soon as a horse displayed a decrease in performance quality, or early signs of lameness, a whole‐body analysis that includes assessment of both conformational susceptibilities and posture/movement during work would be undertaken. Thus, any issues could be compensated for or corrected through individualized physical therapy before major tissue damage has occurred. This “prehabilitation” is highly effective and has become the standard for human athletes.

It should be emphasized that while this type of therapy has great potential, by definition, it is not “one size fits all” (i.e., do this one standardized exercise and the horse is fixed). Rather, just like successful physical/occupational therapy in humans, it requires involvement of trainers and owners working under supervision over a period of time to tailor the mechanical therapy to each horse's specific needs in the context of its athletic function. In particular, a treatment regimen based upon a whole‐body analysis requires the working knowledge of how to establish correct postures within the movements required for the various equine sports. This knowledge is available, as it is the basis for effective training. The question is whether the knowledge and supervision will be primarily provided by veterinarians or by an outside group of professionals. Regardless, as is the case in human orthopedics, veterinarians treating musculoskeletal diseases like navicular syndrome need to have a good understanding of the pathogenesis of mechanical injury in order to fully educate clients about therapeutic options. This understanding should include knowledge of the effects of poor working posture, as well as conformation, and that the primary site of mechanical dysfunction is often not where the tissue lesions have formed.

It should also be stressed that if bad posture/movement continues, therapies that do not address the primary mechanical dysfunction will eventually fail and the disease will progress.^99^ The mechanical environment is critical to the healthy functioning of cells and tissues, and regenerative treatments are likely to also eventually fail if the regenerated cells/tissues are exposed to the same toxic mechanical stresses that damaged the original tissues. Addressing the root cause of mechanical dysfunction is the only way to ensure that the effectiveness of regenerative therapies is maximized and that the careful tissue repair work of the surgeon does not go to waste.

## 7 CONCLUSIONS

A functional perspective based upon insights from comparative anatomy revealed that the biomechanical and pathological features of navicular syndrome are compatible with the failure of an interconnected set of structures adapted for the dispersal of mechanical forces: the navicular enthesis organ. Consideration of the navicular apparatus as an enthesis organ provides an explanation for the tissue lesions and why they vary between cases. It also indicates that tissue damage is primarily due to chronic compression of the navicular enthesis organ that, over time, results in adaptive failure. Exactly where lesions form can vary because the mechanical stress is dispersed across several different tissues and structures throughout the navicular enthesis organ. In addition, chronic generalized compression from overloading can explain the presence of compensatory lesions like enthesophytes in the distal sesamoidean (impar) and collateral ligaments that are often associated with navicular syndrome, but do not always correlate with the degree of lameness.

A comparative perspective also reveals marked similarities between navicular syndrome and chronic osteoarthritis in humans. They both have a primary mechanical pathogenesis, tissue lesions are virtually identical, and many of the same therapeutic approaches have been used. In human medicine, mechanically based therapies, often first developed by trainers and physical therapists, have been subjected to professional scrutiny. Effective biomechanical therapies have thus become widely accepted and are often employed as the treatments of choice. The use of whole‐body mechanical assessment and the success of the related biomechanical therapies have led to the rapid expansion of sports/occupational medicine focused on preventing and treating human athletic injury and osteoarthritis through the use of individually targeted exercises. In contrast, such therapies are generally not used in veterinary medicine beyond corrective shoeing. But the principles developed with humans in mind are broadly applicable to horses as well as other species. Adopting this approach may enable us to improve the therapies available for treating equine diseases like navicular syndrome that are often recurrent and have a poor prognosis in the long term.

Although these therapies have largely not been developed by surgeons, their use is relevant to the field of veterinary surgery. They require that surgeons work closely with owners, trainers, and riders who are acquainted with the animal and how it is habitually worked to decide at what point surgery is actually needed, or to suggest physical or movement therapies as a follow‐up to surgery. Such cooperative strategies in human medicine have proven to be very effective and have directly led to more successful surgical outcomes.

## AUTHORS CONTRIBUTION

Michelle L. Osborn: Substantial contributions to the conception or design of the work; Acquisition, analysis, or interpretation of data for the work; Drafting the work or revising it critically for important intellectual content; Final approval of the version to be published; Agreement to be accountable for all aspects of the work in ensuring that questions related to the accuracy or integrity of any part of the work are appropriately investigated and resolved. Jean Luc Cornille: Acquisition, analysis, or interpretation of data for the work; Drafting the work or revising it critically for important intellectual content; Final approval of the version to be published; Agreement to be accountable for all aspects of the work in ensuring that questions related to the accuracy or integrity of any part of the work are appropriately investigated and resolved. Uriel Blas‐Machado: Acquisition, analysis, or interpretation of data for the work; Drafting the work or revising it critically for important intellectual content; Final approval of the version to be published; Agreement to be accountable for all aspects of the work in ensuring that questions related to the accuracy or integrity of any part of the work are appropriately investigated and resolved. Elizabeth W. Uhl: Substantial contributions to the conception or design of the work; Acquisition, analysis, or interpretation of data for the work; Drafting the work or revising it critically for important intellectual content; Final approval of the version to be published; Agreement to be accountable for all aspects of the work in ensuring that questions related to the accuracy or integrity of any part of the work are appropriately investigated and resolved.

## CONFLICT OF INTEREST

The authors declare no conflict of interest related to this report.
